# Optimizing phenobarbital dosing in critically ill patients with refractory and superrefractory status epilepticus using a population pharmacokinetic model

**DOI:** 10.1111/epi.18517

**Published:** 2025-06-26

**Authors:** Maximilian Stoschus, Moritz L. Schmidbauer, Johannes Starp, Stefan Kunst, Georgios Gakis, Michael Paal, Michael Vogeser, Christina Scharf‐Janssen, Uwe Liebchen, Konstantinos Dimitriadis

**Affiliations:** ^1^ Department of Neurology LMU University Hospital, LMU Munich Munich Germany; ^2^ Department of Anesthesiology LMU University Hospital, LMU Munich Munich Germany; ^3^ Department of Neurology Johns Hopkins University School of Medicine Baltimore Maryland USA; ^4^ Institute of Laboratory Medicine LMU University Hospital, LMU Munich Munich Germany

**Keywords:** individualized dosing, intensive care unit, model‐informed precision dosing, neurointensive care, nonlinear mixed effects modeling, therapeutic drug monitoring

## Abstract

**Objective:**

Current weight‐based dosing fails to account for pharmacokinetic variability in refractory and superrefractory status epilepticus (RSE, SRSE). However, understanding pharmacokinetics in critically ill patients with varying degrees of organ dysfunction can improve both safety and efficacy. Hence, this study aims to quantify key pharmacokinetic variabilities to enable individualized dosing in RSE and SRSE.

**Methods:**

Patients with RSE and SRSE admitted to a neurointensive care unit of a tertiary academic center were retrospectively screened for therapeutic drug monitoring (TDM) samples of phenobarbital. Demographics, laboratory data, comedication, and dosing history were collected from electronic health records. Using a nonlinear mixed effects modeling approach via MONOLIX, a population pharmacokinetic model was developed. Optimal dosing regimens were simulated based on estimated parameters, with target attainment calculated for trough plasma phenobarbital concentrations within 18–40 mg/L.

**Results:**

Thirty‐seven patients contributed 301 TDM samples. Oral bioavailability (96%), volume of distribution (V; 34.3 L), and total body clearance (CL; .38 L/h) were consistent with nonintensive care literature data. Ideal body weight (IBW) implemented as allometric scaling was the only significant covariate improving model fit, demonstrating a positive correlation with the required phenobarbital dose. Simulations identified optimal 12‐h dosing strategies. Oral and intravenous dosing showed minor differences in loading doses but identical maintenance doses, with no significant impact on target attainment for both administration methods. As shown by the coefficients of variation (CVs), intensive care patients exhibited high interindividual (81.36% CV on V, 41.36% CV on CL) and interoccasion variability (36.85% CV on CL), resulting in low target attainment in simulated patients (~40%).

**Significance:**

The pharmacokinetic model characterized phenobarbital pharmacokinetics in patients with RSE and SRSE, showing high oral bioavailability and IBW's impact on V and CL. High pharmacokinetic variability led to low target attainment. Model‐informed precision dosing might improve target attainment in the future.

**Key points**

- Phenobarbital was best described by a one‐compartment model with first‐order absorption and elimination, exhibiting 96% bioavailability.
- IBW significantly affected the volume of distribution and total body clearance.
- IBW‐based optimal dosing for oral and intravenous phenobarbital showed no significant difference in required dosing or target attainment.
- Phenobarbital exhibited high interindividual and interoccasion variability, resulting in low target attainment in intensive care patients.
- The high pharmacokinetic variability of phenobarbital underscores the need for therapeutic drug monitoring in clinical practice.

## 1 INTRODUCTION

Status epilepticus (SE) is a life‐threatening neurologic emergency characterized by prolonged epileptic seizure, lasting >5 min for tonic–clonic SE or >10 min for other types.^1^, ^2^ Refractory SE (RSE) is described as SE unresponsive to standard antiepileptic drug therapy, and superrefractory SE (SRSE) continues for ≥24 h despite or after termination of general anesthesia.^3^, ^4^ SRSE is associated with high morbidity and mortality, with prolonged seizures risking permanent structural and functional brain damage.^5^, ^6^, ^7^, ^8^

More than a century after its introduction, phenobarbital remains a highly effective treatment for SE.^9^, ^10^, ^11^ The American Epilepsy Society acknowledges phenobarbital as an effective first‐line treatment for convulsive SE, with meta‐analyses suggesting its superiority in halting seizures in SE and ensuring sustained seizure control 24 h after cessation.^11^, ^12^ A recent meta‐analysis showed that barbiturate coma, midazolam, or propofol may be an option for RSE treatment, although few studies offer specific drug recommendations for SRSE, contributing to considerable heterogeneity and gaps in real‐world treatment protocols.^13^, ^14^ Due to complications from prolonged use of continuous infusion of anesthetic drugs, there is increasing interest in nonanesthetic treatment options for RSE.^15^ Notably, phenobarbital has proven effective in benzodiazepine‐resistant SE and SRSE, although its use by clinicians still remains limited.^1^, ^16^, ^17^, ^18^ A survey of intensive care physicians found that 50% ranked phenobarbital as their fifth choice among treatment options for SE, and 25% reported never using it.^18^

Concerns about toxicity, including hypotension, sedation, and respiratory depression, may limit its use.^9^, ^17^, ^19^ Consequently, phenobarbital administration necessitates a careful dosing to balance efficacy and safety.

Therapeutic drug monitoring (TDM) helps maintain trough plasma concentrations within the target range of 10–40 mg/L.^9^ Evidence suggests concentrations > 18 mg/L for seizure control in SRSE increase the likelihood of termination 10‐fold; concentrations > 70 mg/L are associated with increased toxicity.^9^, ^16^ Achieving therapeutic target levels remains particularly challenging in critically ill patients with RSE and SRSE, as the heterogeneous degrees of organ dysfunction impact key pharmacokinetic parameters.^20^ Absorption may be influenced by delayed gastric emptying, increased gastric residual volumes, and vomiting, and distribution varies with plasma volume, protein binding, and capillary leakage. Metabolism and elimination can be impaired by hepatic or renal dysfunction and drug interactions, increasing pharmacokinetic variability in intensive care.^21^

Population pharmacokinetic modeling can help assess unexplained population variability, and model‐informed precision dosing, that is, model‐based dosing combined with TDM, further optimizes dosing in patients with high interindividual variability.^21^, ^22^ Although extensive data have been published for neonates and children, pharmacokinetic models for adult populations remain limited.^9^, ^23^, ^24^ Importantly, none report on a critically ill cohort with RSE/SRSE.

Therefore, this study aims to develop a population pharmacokinetic model to characterize key pharmacokinetic parameters, quantify pharmacokinetic variability in real‐world data, and thereby facilitate model‐informed precision dosing of phenobarbital in RSE and SRSE.

## 2 MATERIALS AND METHODS

### 2.1 Study design and data acquisition

Patients with RSE or SRSE, available TDM for phenobarbital, age ≥ 18 years, and no history of hypoxic–ischemic encephalopathy with treatment between 2015 and 2024 at the Neurointensive Care Unit of the LMU University Hospital Munich, Germany were included. This study was approved by the institutional ethics committee of Ludwig Maximilian University of Munich, and the requirement for written consent was waived (project number 20–0906). Patient characteristics (age, sex, body weight, height), dosing histories of phenobarbital, clinical parameters, laboratory data, and selected comedication were retrospectively retrieved from health records. Additional patient information was collected (Appendix S1). Ideal body weight (IBW) was calculated using the formula proposed by Brower et al., and the Status Epilepticus Severity Score (STESS) followed Rossetti et al.'s formular.^25^, ^26^ Phenobarbital was administered in doses according to current guidelines, with loading doses of 10–20 mg/kg and maintenance doses of 1–4 mg/kg.^9^, ^27^ For oral application, tablets were dispersed in 5 mL of water to form a suspension, which was then administered via a gastric tube. Intravenous phenobarbital doses were administered with an infusion duration of 5 min. According to our standard operating procedure, there was no difference in dosing between oral and intravenous administration. Routine TDM of trough concentrations was performed on Mondays, Wednesdays, and Fridays. Serum phenobarbital concentrations were measured using a high‐performance liquid chromatography–ultraviolet method with a quantification range of .6–150 mg/L (Appendix S1).

### 2.2 Population pharmacokinetic modeling

Exploratory statistical analysis and graphical analysis were conducted in R (version 4.3.2). The population pharmacokinetic model was developed using a nonlinear mixed effects modeling approach in MONOLIX (version 2024R1, Lixoft). MONOLIX is software used for population pharmacokinetic analysis and nonlinear mixed effects model development by employing the Stochastic Approximation Expectation–Maximization algorithm.

A one‐compartment model with first‐order absorption and first‐order elimination was used as the structural model.^23^, ^24^, ^28^ Various disposition models were tested, incorporating interindividual variability (IIV), interoccasion variability (IOV), and lag time. IOV occasions were defined as the time intervals between consecutive TDM samples, with a median interval of 48 h in our cohort. A lognormal distribution was assumed for both IIV and IOV. Residual unexplained variability was assessed using additive, proportional, and combined variability models. As the majority of the TDMs were taken after the absorption phase of oral medication, estimation of the absorption rate constant (*k*_a_) was not possible. Therefore, *k*_a_ was fixed at 1.9 h^−1^ according to the literature.^29^

After developing the base model, covariates and their effects were tested. These included patient characteristics (age, sex, body weight, height), clinical parameters (renal replacement therapy, reflux), laboratory data (serum creatinine, bilirubin, albumin, alanine and aspartate aminotransferase), and comedication including valproate, phenytoin, metamizole, and cenobamate. Further covariates, including additional comedication, were selected for analysis, of which only a subset is presented here (Appendix S1: Table S1). Missing data for height and weight were imputed using the population median. Continuous covariates were interpolated over time, whereas categorical covariates used the last observation carried forward method. Continuous covariates were centered around the population median for model implementation.

Before incorporating covariates into the model, exploratory graphical and data analyses were performed in MONOLIX to identify potentially relevant covariates. This approach helped avoid arbitrary testing, reducing the risk of false‐positive associations while supporting model parsimony, thereby minimizing the potential for overfitting. Then, categorical covariates were assessed with additive and proportional shift models, whereas continuous covariates were tested with proportional, power, and exponential models. All covariates, except for patient demographics that remained constant over time, were implemented as time‐varying covariates to maximize the precision of their impact on the model throughout the study period. Medications were implemented as time‐varying covariates by day, meaning their presence or absence was recorded for each day of phenobarbital treatment, including the 2 days prior to its initiation, allowing the model to account for changes in drug exposure over time. Stepwise covariate modeling was performed using forward inclusion, whereby covariates were first assessed for statistical significance (*p* < .05), corresponding to a decrease in the objective function value (OFV) of at least 3.84.^30^ Among these, the covariate model associated with the greatest reduction in the OFV was retained in the model for subsequent rounds. Additional covariates were included in successive rounds according to the same principle, with physiological plausibility and relevance considered at each step. Backward elimination was performed by sequentially removing covariates from the model, beginning with the most recently included covariate. Covariates were retained in the model only if their removal resulted in an increase in the OFV greater than 10.83 (*p* < .001), indicating a statistically significant deletion from the model.^30^

Model quality was assessed by verifying the credibility and precision of estimated parameters and examining the OFV. The coefficient of variation (CV) was determined using the SD (Appendix S1: Equation 1). Diagnostic plots included observed plasma concentrations versus individual and population predicted plasma concentrations in a goodness‐of‐fit plot and assessments of model prediction quality through prediction‐corrected visual predictive checks (Appendix S1: Figure S1). Model quality was further assessed using additional criteria and graphical analyses (Appendix S1). A nonparametric bootstrap with 1000 runs was performed to determine model stability and robustness.

### 2.3 Probability of target attainment analysis

Monte Carlo simulations (*n* = 100 000) were performed to determine the optimal dosing strategy based on significant covariates. To achieve this, the final model was scripted in the C++ programming language and imported into R for simulations and visualization using the mrgsolve package (Appendix S1). Initial simulations tested both 12‐h and 24‐h dosing schedules, with loading doses administered at *t* = 0 h and maintenance doses administered every 12 or 24 h, respectively. The 12‐h dosing schedule was selected over the 24‐h dosing schedule for subsequent simulations to minimize peak–trough fluctuations, reducing variations between maximum and minimum plasma concentrations. The simulations were performed using virtual patients based on pharmacokinetic parameters from the model estimations, including IIV and IOV. For loading doses, a range of intravenous and oral phenobarbital doses (100–2500 mg in 100‐mg intervals) were simulated per covariate–dose combination. Predicted minimum plasma concentrations at t = 12 h (*C*_min_) were analyzed to identify the loading doses that optimized seizure control while minimizing toxicity, defined as the highest probability of *C*_min_ in the range of 18–40 mg/L.^9^, ^16^, ^23^, ^24^, ^27^

Optimal intravenous and oral loading doses were applied as initial doses in maintenance dose simulations. Maintenance doses were then simulated using a range of intravenous and oral phenobarbital doses (50–500 mg in 50‐mg intervals) with 10 000 uniquely simulated patients per covariate–dose combination. Plasma concentrations at *t* = 336 h were assessed to represent *C*_min_ at steady state, based on the elimination half‐life of 62.6 h, which was derived from our estimated population‐specific parameters using a formula from literature.^31^ For these *C*_min_ concentrations, optimal maintenance doses for each covariate combination were determined, similarly to loading doses.

## 3 RESULTS

### 3.1 Patient demographics

Baseline patient characteristics are summarized in Table 1. The cohort included 37 patients, of whom 13 (35%) were female. The median age was 63 years, with a median total body weight of 78.5 kg and a median IBW of 68.8 kg. Patient demographics were missing for height (four patients) and weight (two patients). Most patients suffered from SRSE (95%), whereas only two patients were diagnosed with RSE (5%). The median severity of epilepsy measured by the STESS was 5. The median duration of phenobarbital treatment was 16 days, with a median dose of 200 mg. Approximately two thirds of phenobarbital doses were administered orally (66%), with the remaining doses given intravenously. With a total of 301 TDM samples, patients had a median of 6 samples each and a median phenobarbital concentration of 32.8 mg/L.

**TABLE 1:** Demographic, treatment, and laboratory data of patients at day of admission.

Characteristic	*n* (%) or median (5%, 95%)
Demographics	
Patients	37
Male	24 (65)
Female	13 (35)
Age, years	63.0 (24.0, 83.0)
Height, cm	173.0 (161.0, 187.5)
Total body weight, kg	78.5 (51.7, 93.3)
Ideal body weight, kg	68.8 (53.3, 81.9)
Body mass index, kg/m^2^	24.7 (18.4, 30.2)
SE	
RSE	2 (5)
SRSE	35 (95)
STESS	5 (1.8, 6.0)
Phenobarbital treatment	
Total number of doses	1242
Intravenous doses	418 (34)
Oral doses	824 (66)
Dose, mg	200 (100, 600)
Total treatment duration, days	16 (2, 61)
Total number of TDM samples	301
Number of TDM samples per patient	6 (1, 26)
Phenobarbital concentration, mg/L	32.8 (9.5, 65.8)
Laboratory data	
Serum creatinine, mg/dL	.7 (.3, 3.3)
Aspartate aminotransferase, U/L	28.0 (14.0, 133.0)
Alanine aminotransferase, U/L	27.0 (8.0, 250.0)
Estimated glomerular filtration rate, mL/min	102.0 (15.0, 174.0)
Renal replacement therapy	3 (8)

*Note*: Estimated glomerular filtration rate was calculated using the CKD‐EPI (Chronic Kidney Disease Epidemiology Collaboration) formula. Total treatment duration refers to the period between first and last dose of phenobarbital for each patient.

Abbreviations: RSE, refractory SE; SE, status epilepticus; SRSE, superrefractory SE; STESS, Status Epilepticus Severity Score; TDM, therapeutic drug monitoring via single blood sample.

### 3.2 Population pharmacokinetic modeling

The data were best described by a one‐compartment model with first‐order absorption and first‐order elimination with IIV on both volume of distribution (V) and clearance (CL), along with IOV on CL and a proportional residual variability model. The final covariate model included IBW as a covariate through allometric scaling applied to V and CL, centered around the population median IBW (Equations 1 and 2).^30^, ^32^

For Equation 1, *V* = volume of distribution, $\theta_{V}$ = population value of *V*, IBW_i_ = individual ideal body weight, and medIBW = population median of ideal body weight.

(1) $$V_{i}=\theta_{V}\times {\left(\frac{\mathrm{IB}W_{i}}{\text{medIBW}}\right)}^{1}$$

For Equation 2, CL = total body clearance, $\theta_{\mathrm{CL}}$ = population value of CL, IBW_*i*_ = individual ideal body weight, and medIBW = population median of ideal body weight.

(2) $$CL_{i}=\theta_{\mathrm{CL}}\times {\left(\frac{\mathrm{IB}W_{i}}{\text{medIBW}}\right)}^{.75}$$

**FIGURE 1:**
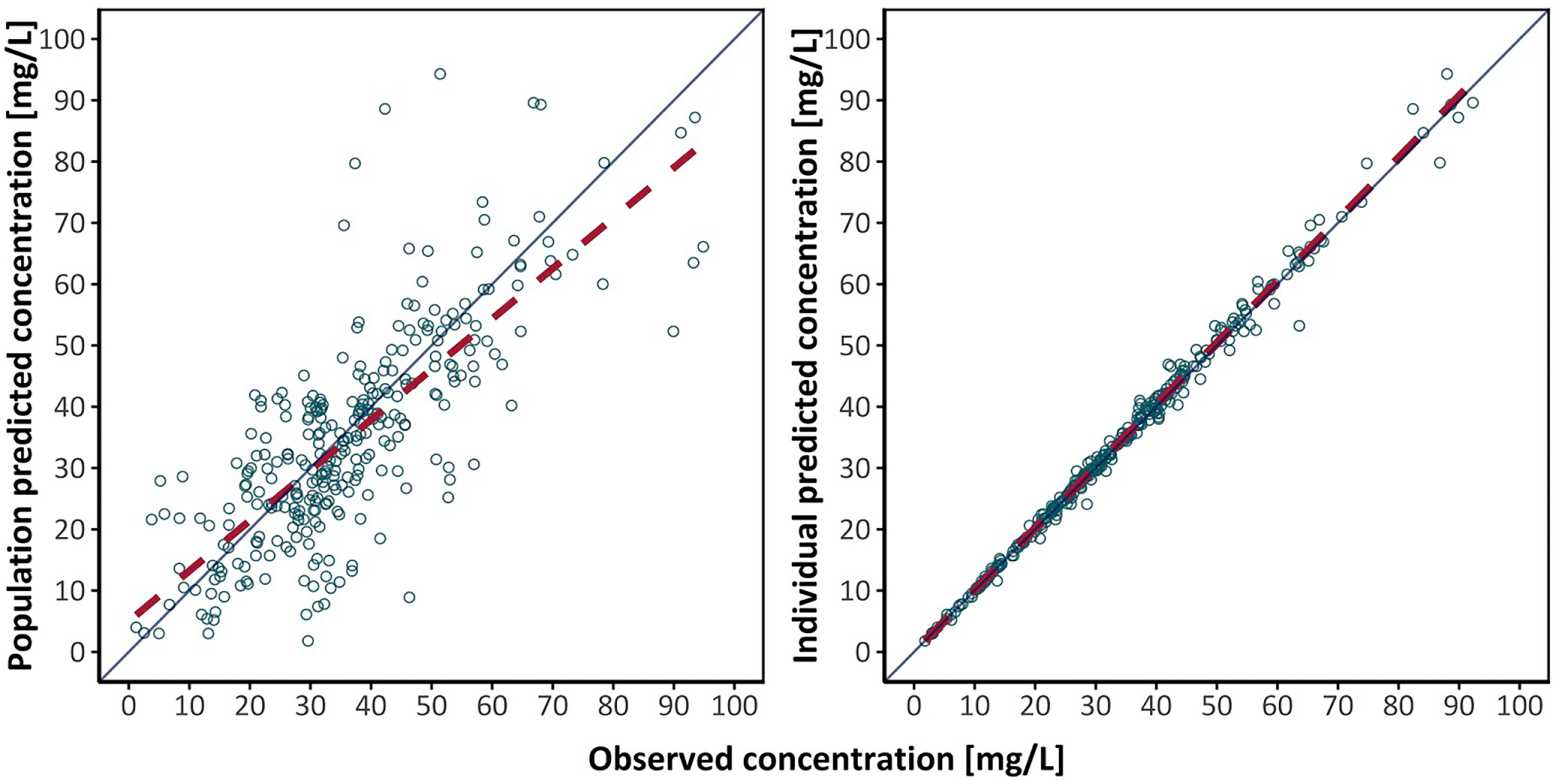
Population predicted concentration and individual predicted concentration of phenobarbital versus observed concentrations of phenobarbital for the final model. The solid lines represent lines of identity. The red dashed lines represent the regression lines. Rings represent observed/predicted plasma concentration.

Compared to the base model, covariate inclusion of IBW substantially lowered the residual unexplained variability (−10.8%) and the OFV by 71.9 (Appendix S1: Table S2). Other covariates did not significantly reduce the OFV, indicating no improvement in the model prediction, and were therefore excluded from the model. Evaluation of the model presented low relative standard errors (RSE < 30%), indicating high confidence in the estimated population parameters. Bioavailability was estimated at F = .96 (Table 2). V was estimated at 34.3 L and CL at .38 L/h, corresponding to .44 L/kg for V and .0048 L/h/kg for CL when adjusted for the population median weight of 78.5 kg. The CVs of IIV on V (81.36%) and CL (41.36%) were high, indicating variability between patients across the population. The CV of the IOV on CL (36.85%) indicated in‐patient variability of CL over time. The nonparametric bootstrap analysis using *n* = 1000 replicate runs demonstrated the robustness of the model, providing parameter estimates consistent with those obtained from the final model. Goodness‐of‐fit plots and individual concentration–time profiles demonstrated adequate representation of measured concentrations by the model (Figure 1, Appendix S1: Figure S2).

**TABLE 2:** Estimated parameters of the population pharmacokinetic model of phenobarbital in critically ill patients (*n*_patients_ = 37).

Parameter	Final model estimate, RSE (%; 95% CI)		Bootstrap median (95% CI)
Fixed‐effects parameters	Value		Value
F	.96 (3.95; .85–.99)		.99 (.84–.99)
*k*_a_, h^−1^ ^a^	1.9		
V, L	34.3 (12.7; 26.81–43.89)		35.2 (25.08–45.98)
CL, L/h	.38 (7.81; .32–.44)		.37 (.32–.43)
IIV parameters	SD	CV %	SD
V, L	.71 (12.1; .56–.9)	81.36	.7 (.37–.96)
CL, L/h	.4 (16.2; .29–.54)	41.36	.39 (.17–.58)
IOV parameters	SD		SD
CL, L/h	.36 (6.64; .31–.41)	36.85	.29 (.19–.4)
RUV parameters	SD		SD
Proportional	.082 (7.76; .071–.096)		.11 (.069–.17)

Abbreviations: CI, confidence interval; CL, total body clearance; CV, coefficient of variation; F, bioavailability; IIV, interindividual variability; IOV, interoccasion variability; *k*_a_, absorption rate constant; RSE, relative standard error; RUV, residual unexplained variability; V, volume of distribution.

^a^ *k*_a_ was fixed according to literature values.

### 3.3 Model‐based dosing recommendations

The 24‐h schedule required larger loading and maintenance doses, causing greater fluctuation in plasma concentrations and a higher risk of toxicity than the 12‐h dosing schedule (Appendix S1: Figure S3). Therefore, simulations were conducted with dosing every 12 h based on IBW. A range of IBWs (55–80 kg in 5‐kg intervals) was tested, ensuring that all selected IBWs fell within the range of the original patient cohort (52.4–82.4 kg).

**FIGURE 2:**
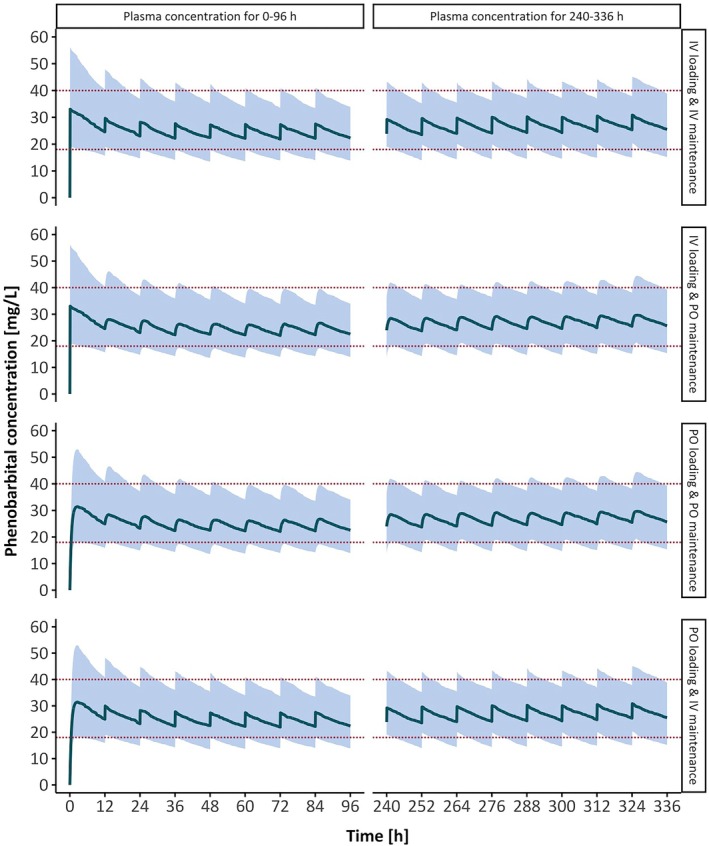
Concentration–time profiles of phenobarbital for an ideal body weight of 70 kg across possible combinations of administration routes (IV, intravenous; PO, oral) for a 12‐h dosing schedule with optimal dosing recommendations, simulated for 1000 patients. Loading dose = 1100 mg, maintenance dose = 150 mg. The solid lines represent the median, the blue shaded areas show the 50% prediction interval, and the dotted red lines indicate the target range (18–40 mg/L).

Higher IBWs required increased dosing for both loading and maintenance doses to achieve target concentrations. When comparing intravenous and oral administration for loading doses, only minor differences in the optimal dosing amounts were observed. For maintenance doses, optimal amounts were identical for both intravenous and oral administration, regardless of the loading dose route (Table 3). Intravenous and oral administration of phenobarbital resulted in similar concentration–time profiles (Figure 2).

**TABLE 3:** Optimal dosing regimens for a 12‐h dosing schedule for phenobarbital across different IBWs for minimum concentrations of 18–40 mg/L.

Covariate	Loading dose, mg		Maintenance dose, mg
IBW, kg	Intravenous	Oral	Intravenous/oral
55	1000	900	100
60	1000	1000	150
65	1000	1000	150
70	1100	1100	150
75	1300	1300	150
80	1300	1300	150

*Note*: Intravenous doses were administered as short infusions with an infusion duration of 5 min. Simulations were executed 100 000 times for each loading dose and IBW combination, and 10 000 times for each maintenance dose and IBW combination. Loading dose: *n*_simulated patients_ = 30 000 000; maintenance dose: *n*_simulated patients_ = 2 400 000.

Abbreviation: IBW, ideal body weight.

**FIGURE 3:**
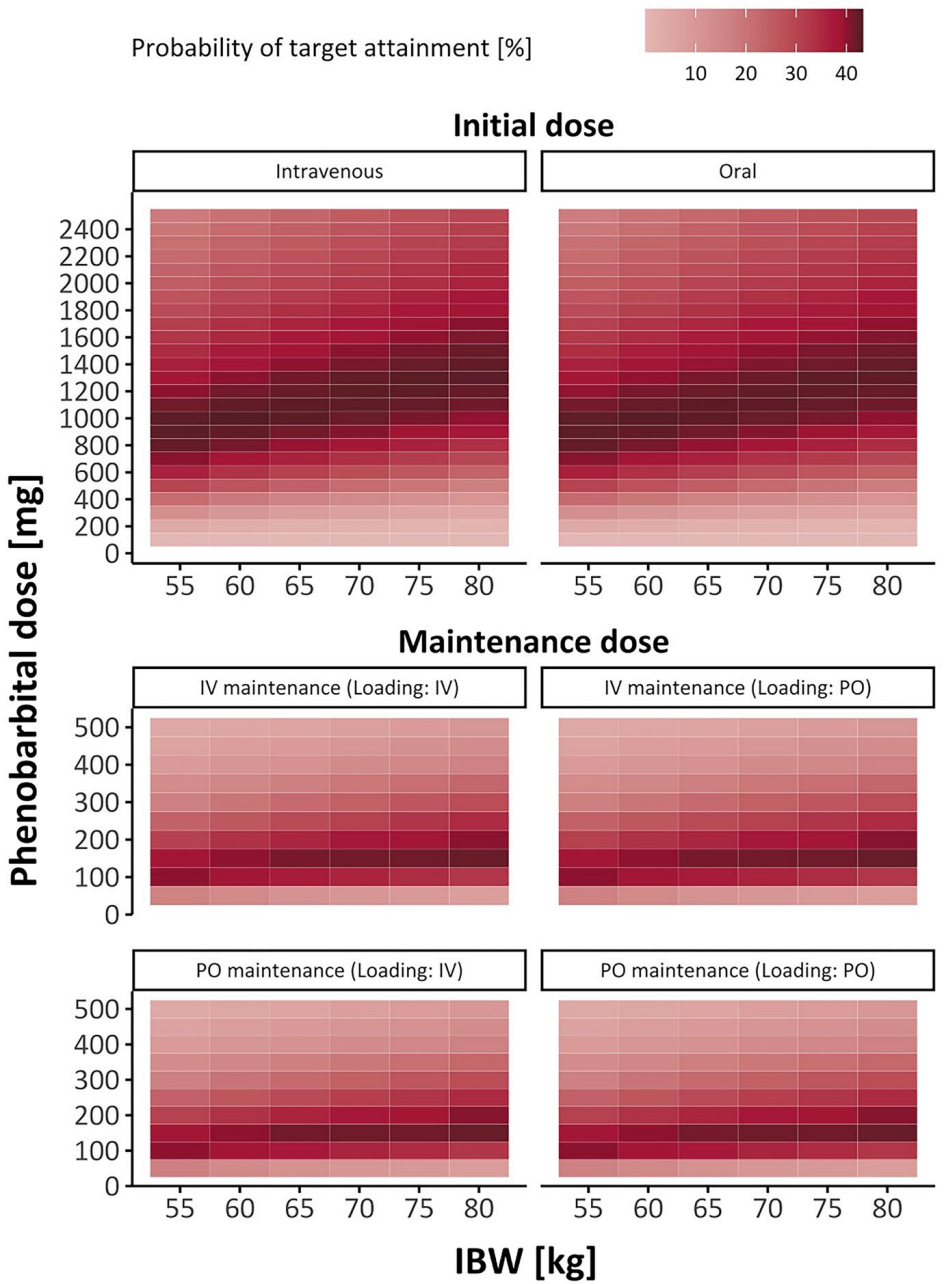
Probability of target attainment for intravenous and oral loading and maintenance doses in simulated patients, based on dose and ideal body weight (IBW). The target range for *C*_min_ was 18–40 mg/L. Simulated concentrations were observed at *t* = 12 h for initial doses and *t* = 336 h for maintenance doses. IV, intravenous; PO, oral.

The percentage of patients attaining target at 12 h was 42.7% for intravenous and 42.5% for oral loading doses across all IBWs. For maintenance doses, the percentages for steady state concentrations (*t* = 336 h) were 44.3% for intravenous and 44.5% for oral administration, regardless of the loading dose application form (Figure 3).

## 4 DISCUSSION

Our pharmacokinetic model showed high bioavailability of oral phenobarbital in intensive care patients, resulting in negligible differences between oral and intravenous dosing regimens. Overall, phenobarbital exhibited high pharmacokinetic variability, which was only partly explainable by IBW. This resulted in low empirical target attainment, emphasizing the need for TDM integration or individualized model‐based dosing strategies in clinical practice.

It is a strength of our study that we could estimate bioavailability based on data after parenteral and oral dosing (F = 96%), which was so far unreported in adult intensive care unit (ICU) patients. Our findings align with the study of Nelson et al., who reported a similar bioavailability of 94.9% in six healthy adult volunteers.^33^ Comparable studies reported similar population parameter estimates for CL, such as Vučićević et al. (CL/F = .314 L/h) and Teixeira‐da‐Silva et al. (CL/F = .236 L/h).^23^, ^24^ However, in both cases, CL could not be separated from bioavailability, and V was fixed due to estimation limitations.

IBW significantly influenced V and CL, improving model fit. This aligns with previous studies and a meta‐analysis identifying body weight as a key covariate.^9^, ^23^, ^24^, ^34^, ^35^ Other covariates, such as comedication, were found to be insignificant in our population pharmacokinetic model. It should be acknowledged that previous studies reported reduced CL with concomitant therapy with phenytoin and valproic acid.^23^, ^24^, ^34^ Given the common coadministration of other antiepileptics in our cohort, such effects cannot be ruled out and might be included in the estimated CL parameter.

Simulations were conducted using a 12‐h dosing schedule, as the simulation of a 24‐h schedule suggested both higher loading and maintenance doses. These increased doses resulted in higher variability in plasma levels, with predicted plasma concentrations within the 50% prediction interval reaching toxic concentrations of >70 mg/L after the loading dose. Additionally, steady state concentrations were less stable for the maintenance doses in the 24‐h schedule, leading to higher peak‐trough‐fluctuations in predicted plasma levels (Appendix S1: Figure S3). Hence, despite phenobarbital's long half‐life, a 12‐h schedule offers a more stable dosing regimen.

The dosing recommendations (Table 3) are designed for ease of use in daily clinical practice. The appropriate dose can be selected based on the patient's IBW, with adjustments made for the method of administration (oral or intravenous). These recommendations provide a practical approach to determine both loading and maintenance doses, particularly in situations where clinicians have limited patient‐specific data available. This enables informed dosing decisions that align with pharmacokinetic principles and support optimal target attainment. The IBW‐based dosing recommendations for the proposed target plasma levels are comparable with the standard adult dosing of 10–20 mg/kg or 15–20 mg/kg for loading and 1–4 mg/kg for maintenance.^9^, ^27^ However, it is important to note that standard dosing is based on total body weight, whereas our dosing recommendations refer to IBW. IBW might better reflect physiological factors like organ function and distribution, which do not always correlate with changes in total body weight. Although existing dosing recommendations in the literature are based solely on intravenous phenobarbital, we found that oral dosing amounts are nearly identical. Consequently, based on our results, oral administration of phenobarbital serves as an alternative to intravenous administration in intensive care. Oral maintenance doses might be advantageous for transitioning patients to a rehabilitation program that includes phenobarbital remission therapy, which necessitates eliminating dependence on intravenous application. In cases of parenteral administration, it is important to note that extremely high loading doses should be administered slowly (maximum 100 mg/min) and with close monitoring of vital signs in an ICU setting, as high doses require close monitoring, including readiness for intubation.^9^, ^17^, ^27^ Additionally, lowering the infusion rate can help reduce the risk of respiratory depression and hypotension.^19^

We found high between‐ and in‐patient variability, resulting in a relatively low probability of achieving target plasma levels with empirical dosing. Although IIV on V was either fixed or associated with a high relative standard error in comparable studies, our data allowed for precise estimation of IIV on V with a low relative standard error, demonstrating robust parameter estimation.^23^, ^24^, ^28^, ^34^, ^35^ Even though one study reported slightly higher IIV for CL, another estimated only half of the IIV observed in our model.^23^, ^24^ The high IIV for CL in our model may have resulted from the inclusion of exclusively ICU patients, reflecting high pharmacokinetic variability commonly observed in intensive care.^21^

A potential strategy to improve target attainment and encourage the use of phenobarbital could be the implementation of model‐informed precision dosing, with our model as a candidate model. It is important to note that an external evaluation should be done prior to clinical use of our model as recommended.^36^ This approach uses pharmacokinetic models and software tools to individualize dosing based on patient characteristics, with TDM samples incorporated using the Bayesian principle.^37^ This enables real‐time TDM data to be continuously integrated into the dosing software, allowing for ongoing updates of drug concentration predictions and the refinement of dosing recommendations over time, thereby increasing the likelihood of achieving and maintaining therapeutic levels in clinical practice.^37^ By optimizing dosing and minimizing side effects, this could ultimately elevate the role of phenobarbital in clinical use.

Although this population pharmacokinetic model presents valuable insights into dosing of phenobarbital in ICU patients, certain limitations should be acknowledged.

Insufficient absorption‐phase data prevented precise estimation of the absorption rate constant.

Given that Viswanathan et al.^29^ specifically studied the bioavailability and absorption rate of oral phenobarbital, capturing samples within the absorption phase, we considered their absorption rate appropriate for our model.

The study shows a limited sample size and an extended inclusion period, both of which reflect the rarity and complexity of RSE and SRSE. These conditions inherently restrict the number of eligible patients for inclusion. Nonetheless, nonlinear mixed effects modeling, particularly developed for sparse data, is well‐suited for the development of pharmacokinetic models when only few observations are available.^30^ Populations in which data collection is particularly challenging are often those in greatest need of precise and individualized dosing.^30^

Furthermore, the present cohort might have limited the ability to identify covariates.^23^, ^24^ However, our dataset presented relatively rich sampling, with a median of six TDM samples per patient, nearly three times the number of samples per individual compared to the other studies.^23^, ^24^ This allowed a crucial assessment of between‐ and in‐patient variability throughout phenobarbital treatment, capturing variability in intensive care patients well.

The range of IBWs for simulations and optimal dosing recommendations was restricted, and extrapolation beyond this range could have led to misleading conclusions. However, our cohort represents a typical IBW range also present in other ICUs, with most patient characteristics likely falling within this range.

This model was developed exclusively for adult patients and is not intended for pediatric use. Population parameters and dosing recommendations are based on adult‐specific physiology and their IBW. Extrapolation beyond this population is not appropriate, which is in line with standard practices in pharmacokinetic modeling. Nevertheless, extensive data on neonates and children have already been published.

## 5 CONCLUSIONS

Our population pharmacokinetic model characterized the pharmacokinetics of phenobarbital in patients with RSE and SRSE, demonstrating almost complete oral bioavailability in intensive care patients while highlighting the influence of IBW on V and CL. Interindividual and interoccasion variabilities were high, indicating substantial pharmacokinetic variability in intensive care patients. Simulations showed a low probability of target attainment employing dosing based on IBW. Therefore, model‐informed precision dosing is recommended as a valuable approach to improve target attainment.

## AUTHOR CONTRIBUTIONS

**Maximilian Stoschus:** Data curation; formal analysis; investigation; methodology; resources; software; validation; visualization; writing—original draft preparation (lead), review and editing. **Uwe Liebchen:** Conceptualization; methodology; project administration; resources; software; supervision; writing—review and editing (lead). **Moritz L. Schmidbauer:** Conceptualization; data curation; investigation; project administration; resources; supervision; writing—review and editing. **Johannes Starp:** Data curation; formal analysis; methodology; software; writing—review and editing. **Konstantinos Dimitriadis:** Conceptualization; project administration; supervision; writing—review and editing. **Stefan Kunst and Georgios Gakis:** Data curation; investigation; resources. **Michael Paal and Michael Vogeser:** Resources; writing—review and editing. **Christina Scharf‐Janssen:** Supervision; writing—review and editing.

## CONFLICT OF INTEREST STATEMENT

U.L. reports consulting fees from CytoSorbents Europe and Medows Sarl, and he participated on an advisory board for Roche Diagnostics International. C.S.‐J. has received speaker honoraria from CytoSorbents Europe. The remaining authors have no conflicts of interest. We confirm that we have read the Journal's position on issues involved in ethical publication and affirm that this report is consistent with those guidelines.

## Supporting information


Appendix S1.

## Data Availability

The data that support the findings of this study are available from the corresponding author upon reasonable request.
