# Supplementary material for: Optimizing phenobarbital dosing in critically ill patients with refractory and superrefractory status epilepticus using a population pharmacokinetic model
Source: Epilepsia. 2025 Jun 26;66(10):3757–68. doi: 10.1111/epi.18517 (PMC12605696; doi:10.1111/epi.18517)
Supplement: Supplementary file 1 — Appendix S1. [file EPI-66-3757-s001.docx]

***Supplementary material***

Optimising phenobarbital dosing in critically ill patients with refractory and super-refractory status epilepticus using a population pharmacokinetic model

Maximilian Stoschus^1,2^, Moritz L. Schmidbauer^1^, Johannes Starp^2^, Stefan Kunst^1^, Georgios Gakis^1,4^, Michael Paal^3^, Michael Vogeser^3^, Christina Scharf-Janssen^2^, Uwe Liebchen^2,#,*^, Konstantinos Dimitriadis^1,#^

^1^Department of Neurology, LMU University Hospital, LMU Munich, Germany

^2^Department of Anaesthesiology, LMU University Hospital, LMU Munich, Germany

^3^Institute of Laboratory Medicine, LMU University Hospital, LMU Munich, Germany

^4^Department of Neurology, Johns Hopkins University School of Medicine, Baltimore, MD, United States

^#^Shared last authorship

*Corresponding author: Dr. med. Uwe Liebchen, Department of Anaesthesiology, LMU University Hospital, LMU Munich, Marchioninistrasse 15, 81377 Munich, Germany, Tel +49 89 4400 1681160, E‑mail: [uwe.liebchen@med.uni-muenchen.de](mailto:uwe.liebchen@med.uni-muenchen.de)

Table of Contents

[1 Supplementary materials and methods 3](#_Toc193319321)

[1.1 Collected demographics, clinical parameters, laboratory data and co-medication for covariate assessment 3](#_Toc193319322)

[1.1.1 Demographics 3](#_Toc193319323)

[1.1.2 Clinical parameters 3](#_Toc193319324)

[1.1.3 Laboratory data 3](#_Toc193319325)

[1.1.4 Co-medication 3](#_Toc193319326)

[1.2 Analytical method for phenobarbital quantification in the laboratory 3](#_Toc193319327)

[1.3 Equation for the calculation of coefficient of variation (CV) 4](#_Toc193319328)

[1.4 Population pharmacokinetic modeling: Additional criteria and graphical analyses 4](#_Toc193319329)

[1.5 Probability of target attainment analysis: C++ model script for “mrgsolve” 4](#_Toc193319330)

[2 Supplementary figures 7](#_Toc193319331)

[2.1 Prediction-corrected visual predictive checks (pcVPC) 7](#_Toc193319332)

[2.2 Individual concentration-time profiles 8](#_Toc193319333)

[2.3 Concentration-time profiles for simulated 12-hour and 24-hour schedules 9](#_Toc193319334)

# Supplementary materials and methods

## Collected demographics, clinical parameters, laboratory data and co-medication for covariate assessment

### Demographics

- Sex, age, height, weight, body mass index, ideal body weight
- Demographic variables were collected at the start of phenobarbital treatment, considered continuous, and did not change over time.

### Clinical parameters

- Reflux, renal replacement therapy, nasogastric tube, jejunal tube
- Clinical parameters were documented for each phenobarbital treatment day and considered time-varying covariates. Reflux was treated as a continuous variable, while renal replacement therapy, nasogastric tube and jejunal tube were binary.

### Laboratory data

- Urea, serum creatinine, glomerular filtration rate (CKD-EPI: chronic kidney disease epidemiology collaboration), C-reactive protein (CRP), albumin, bilirubin, aspartate aminotransferase, alanine aminotransferase, gamma-glutamyltransferase, lactate dehydrogenase
- Laboratory data were documented for each phenobarbital treatment day and considered continuous, time-varying covariates.

### Co-medication

- Co-medications were recorded for each phenobarbital treatment day and two days prior to its initiation. They were treated as binary, time-varying covariates on a daily basis.
- Atorvastatin, rosuvastatin, simvastatin, furosemide, torasemide, hydrochlorothiazide, spironolactone, esomeprazole, pantoprazole, metoclopramide, clobazam, clonazepam, diazepam, lorazepam, midazolam, oxazepam, zolpidem, zopiclone, perampanel, valproate, phenytoin, brivaracetam, gabapentin, lamotrigine, cenobamate, pregabalin, topiramate, zonisamide, olanzapine, quetiapine, risperidone, haloperidol, etacrynic acid, melperone, pipamperone, sufentanil, fentanyl, codeine, morphine, pethidine, piritramide, ketamine, isoflurane, levetiracetam, lacosamide, metamizole, naloxone (intravenous), naloxone (oral), paracetamol, perenterol, propranolol, propofol, thiopental, valsartan

## Analytical method for phenobarbital quantification in the laboratory

- Whole blood tubes were centrifuged at 2500 g for 10 minutes at room temperature. Phenobarbital in serum was quantified by HPLC-UV with a commercially available IVD kit from Chromsystems (Gräfelfing, Germany) according to the manufacturer’s instruction. The linear calibration range was 0.6 – 150 mg/L. Briefly, 50 µl sample (either calibrator, quality control, patient specimen) were mixed with 75 µL of internal standard, followed by the addition of 25 µL precipitation reagent and centrifugation at 10000 g for 10 minutes at room temperature. The supernatant was then diluted 1:2 with stabilization buffer in a glass vial that was loaded to an autosampler ready for injection of 20 µL. Analysis was done with an Alliance HPLC system with UV detection at 204 nm. Quality control samples were included with each run, yielding coefficients of variation of 0.8%, 1.1%, and 1.5% at phenobarbital concentrations of 11, 17, and 22 mg/L, respectively.

## Equation for the calculation of coefficient of variation (CV)

Equation 1: Formula for calculation of coefficient of variation (% CV) for interindividual variability (IIV) and interoccasion variability (IOV). The standard deviation is represented by $\omega$.

$$\%CV=\sqrt{e^{\omega^{2}}-1}*100\%$$

## Population pharmacokinetic modeling: Additional criteria and graphical analyses

- Further criteria and graphical analysis for assessment of model quality included Akaike Information Criterion (AIC), (corrected) Bayesian Information Criterion (BIC/BICc), individual-predicted plasma concentrations (IPRED) versus individual weighted residuals (IWRES) and distribution of the normalised prediction distribution errors (NPDE).

## Interacting medication per patient

Table 1: Overview of potentially interacting medications administered per patient during phenobarbital treatment and in the two days prior to its initiation.

| **Medication** | **Number of patients (%)** |
| --- | --- |
| Furosemide | 36 (97) |
| Lacosamide | 35 (95) |
| Metamizole | 35 (95) |
| Levetiracetam | 34 (92) |
| Pantoprazole | 34 (92) |
| Sufentanil | 33 (89) |
| Midazolam | 30 (81) |
| Paracetamol | 30 (81) |
| Propofol | 29 (78) |
| Metoclopramide | 24 (65) |
| Naloxone oral | 24 (65) |
| Lorazepam | 22 (59) |
| Piritramide | 16 (43) |
| Esomeprazole | 15 (41) |
| Clobazam | 14 (38) |
| Atorvastatin | 11 (30) |
| Perampanel | 11 (30) |
| Quetiapine | 11 (30) |
| Hydrochlorothiazide | 10 (27) |
| Etacrynic acid | 10 (27) |
| Movicol | 9 (24) |
| Valproate | 9 (24) |
| Phenytoin | 8 (22) |
| Codeine | 6 (16) |
| Topiramate | 6 (16) |
| Fentanyl | 5 (14) |
| Morphin | 5 (14) |
| Melperon | 4 (11) |
| Brivaracetam | 3 (8) |
| Diazepam | 3 (8) |
| Torasemide | 3 (8) |
| Zonisamide | 3 (8) |
| Gabapentin | 2 (5) |
| Haloperidol | 2 (5) |
| Lamotrigine | 2 (5) |
| Loperamide | 2 (5) |
| Oxazepam | 2 (5) |
| Pethidine | 2 (5) |
| Rosuvastatin | 2 (5) |
| Simvastatin | 2 (5) |
| Spironolactone | 2 (5) |
| Zolpidem | 2 (5) |
| Zopiclone | 2 (5) |
| Acetazolamide | 1 (3) |
| Cenobamate | 1 (3) |
| Clonazepam | 1 (3) |
| Isoflurane | 1 (3) |
| Ketamine | 1 (3) |
| Naloxone intravenous | 1 (3) |
| Olanzapine | 1 (3) |
| Perenterol | 1 (3) |
| Pregabalin | 1 (3) |
| Pipamperone | 1 (3) |
| Propranolol | 1 (3) |
| Risperidone | 1 (3) |
| Thiopental | 1 (3) |
| Valsartan | 1 (3) |

## Model development process by round

- The final model showed an objective function value of OFV = 1813.02. The Akaike information criteria (AIC = 1827.02), Bayesian information criteria (BIC = 1852.97) and corrected Bayesian information criteria (BICc = 1848.78) were also significantly lower in the final model compared to the base model.

Table 2: Overview of model development: Likelihood, covariate functions, affected pharmacokinetic parameters and covariate inclusion decisions.

| **Model** | **COV** | **Parameter** | **COV function** | **Likelihood** | | | | **Inclusion** |
| --- | --- | --- | --- | --- | --- | --- | --- | --- |
|  |  |  |  | OFV | AIC | BIC | BICc |  |
| Base model |  |  |  | 1884.92 | 1898.92 | 1924.87 | 1920.68 |  |
| **Round 1:** (Reference: Base model) | |  |  |  |  |  |  |  |
| Base model + RRT | RRT | CL | ADD | 2069.71 | 2085.71 | 2115.36 | 2111.17 | negative |
| Base model + RRT | RRT | CL | PROP | 2020.27 | 2036.27 | 2065.93 | 2061.74 | negative |
| Base model + TBW | TBW | CL | EXP | 1882.83 | 1898.83 | 1928.49 | 1922.20 | negative |
| Base model + TBW | TBW | V | EXP | 1772.24 | 1788.24 | 1817.9 | 1811.61 | negative, RSE > 30 |
| Base model + TBW | TBW | CL | POWER | 1883.70 | 1899.70 | 1929.36 | 1923.07 | negative |
| Base model + TBW | TBW | V | POWER | 1950.27 | 1966.27 | 1995.93 | 1989.64 | negative |
| Base model + IBW | IBW | CL | EXP | 1988.40 | 2004.40 | 2034.06 | 2027.77 | negative |
| Base model + IBW | IBW | V | EXP | 1991.50 | 2007.50 | 2037.15 | 2030.86 | negative |
| Base model + IBW | IBW | CL | POWER | 1740.43 | 1756.43 | 1786.08 | 1779.79 | negative, RSE > 30 |
| Base model + IBW | IBW | V | POWER | 1989.14 | 2005.14 | 2034.79 | 2028.51 | negative |
| Base model + HT | HT | V | EXP | 1773.10 | 1789.10 | 1818.75 | 1812.46 | negative, RSE > 30 |
| Base model + HT | HT | V | POWER | 1956.03 | 1972.03 | 2001.69 | 1995.40 | negative |
| Base model + CREA | CREA | CL | EXP | 2054.75 | 2070.75 | 2100.41 | 2096.21 | negative |
| Base model + CREA | CREA | CL | POWER | 1842.34 | 1858.34 | 1887.99 | 1883.80 | negative, RSE > 30 |
| Base model + BILI | BILI | V | EXP | 2060.63 | 2076.63 | 2106.29 | 2102.09 | negative |
| Base model + BILI | BILI | V | POWER | 1946.08 | 1962.08 | 1991.74 | 1987.54 | negative |
| Base model + AGE | AGE | CL | EXP | 1916.55 | 1932.55 | 1962.20 | 1955.92 | negative |
| Base model + AGE | AGE | V | EXP | 1953.84 | 1969.84 | 1999.49 | 1993.21 | negative |
| Base model + AGE | AGE | CL | POWER | 2024.09 | 2040.09 | 2069.75 | 2063.46 | negative |
| Base model + AGE | AGE | V | POWER | 1886.85 | 1902.85 | 1932.51 | 1926.22 | negative |
| Base model + BMI | BMI | CL | EXP | 1916.54 | 1932.54 | 1962.20 | 1955.91 | negative |
| Base model + BMI | BMI | V | EXP | 1993.20 | 2009.20 | 2038.85 | 2032.57 | negative |
| Base model + BMI | BMI | CL | POWER | 2025.93 | 2041.93 | 2071.59 | 2065.30 | negative |
| Base model + BMI | BMI | V | POWER | 1990.11 | 2006.11 | 2035.76 | 2029.47 | negative |
| Base model + ALB | ALB | V | EXP | 1836.01 | 1852.01 | 1881.66 | 1877.47 | negative, implausible relation |
| Base model + ALB | ALB | V | POWER | 2019.12 | 2035.12 | 2064.78 | 2060.58 | negative |
| Base model + MDZ | MDZ | CL | ADD | 2014.97 | 2030.97 | 2060.63 | 2056.43 | negative |
| Base model + MDZ | MDZ | CL | PROP | 2054.06 | 2070.06 | 2099.72 | 2095.53 | negative |
| Base model + TBW | TBW | V, CL | ALLO | 1985.38 | 1999.38 | 2025.33 | 2021.14 | negative |
| Base model + TBW | TBW | V theta,  CL allometric | ALLO | 1917.23 | 1933.23 | 1962.89 | 1958.70 | negative |
| Base model + TBW | TBW | V allometric,  CL theta | ALLO | 1845.55 | 1861.55 | 1891.20 | 1887.01 | negative,  RSE > 30 |
| Base model + HT | HT | V, CL | ALLO | 1847.39 | 1861.39 | 1887.34 | 1883.15 | positive, IBW better |
| Base model + HT | HT | V theta,  CL allometric | ALLO | 1910.14 | 1926.14 | 1955.80 | 1951.61 | negative |
| Base model + HT | HT | V allometric,  CL theta | ALLO | 2016.94 | 2032.94 | 2062.60 | 2058.41 | negative |
| Base model + BMI | BMI | V, CL | ALLO | 1851.00 | 1865.00 | 1890.95 | 1886.76 | Positive, IBW better |
| Base model + BMI | BMI | V theta,  CL allometric | ALLO | 2020.52 | 2036.52 | 2066.17 | 2061.98 | negative |
| Base model + BMI | BMI | V allometric,  CL theta | ALLO | 1987.65 | 2003.65 | 2033.31 | 2029.12 | negative |
| Base model + IBW | IBW | V, CL | ALLO | 1813.02 | 1827.02 | 1852.97 | 1848.78 | positive, best model |
| Base model + IBW | IBW | V theta,  CL allometric | ALLO | 2056.70 | 2072.70 | 2102.35 | 2098.16 | negative |
| Base model + IBW | IBW | V allometric,  CL theta | ALLO | 1918.13 | 1934.13 | 1963.79 | 1959.60 | negative |
| **Round 2:** (Reference: Base model + IBW (allometric)) | |  |  | 1813.02 | 1827.02 | 1852.97 | 1848.78 |  |
| Base model + IBW + AGE | AGE | V | POWER | 1951.63 | 1967.63 | 1997.28 | 1993.09 | negative |
| Base model + IBW + AGE | AGE | V | EXP | 1919.48 | 1935.48 | 1965.14 | 1960.65 | negative |
| Base model + IBW + AGE | AGE | V | PROP | 1916.91 | 1932.91 | 1962.57 | 1958.38 | negative |
| Base model + IBW + AGE | AGE | CL | POWER | 1980.92 | 1996.92 | 2026.58 | 2022.39 | negative |
| Base model + IBW + AGE | AGE | CL | EXP | 1878.37 | 1894.37 | 1924.02 | 1919.83 | negative |
| Base model + IBW + AGE | AGE | CL | PROP | 1914.60 | 1930.60 | 1960.26 | 1956.07 | negative |
| Base model + IBW + ALB | ALB | V | POWER | 1878.65 | 1894.65 | 1924.30 | 1920.11 | negative |
| Base model + IBW + ALB | ALB | V | EXP | 1908.79 | 1924.79 | 1954.45 | 1950.26 | negative |
| Base model + IBW + ALB | ALB | V | PROP | 1875.32 | 1891.32 | 1920.98 | 1916.78 | negative |
| Base model + IBW + BILI | BILI | V | POWER | 1805.06 | 1821.06 | 1850.72 | 1846.53 | negative, RSE > 30 |
| Base model + IBW + BILI | BILI | V | EXP | 1880.82 | 1896.82 | 1926.48 | 1922.29 | negative |
| Base model + IBW + BILI | BILI | V | PROP | 1948.22 | 1964.22 | 1993.88 | 1989.69 | negative |
| Base model + IBW + AST | AST | V | POWER | 2054.26 | 2070.26 | 2099.92 | 2095.73 | negative |
| Base model + IBW + AST | AST | V | EXP | 1946.52 | 1962.52 | 1992.18 | 1987.98 | negative |
| Base model + IBW + AST | AST | V | PROP | 1982.14 | 1998.14 | 2027.79 | 2023.60 | negative |
| Base model + IBW + ALT | ALT | V | POWER | 1986.62 | 2002.62 | 2032.27 | 2028.08 | negative |
| Base model + IBW + ALT | ALT | V | EXP | 1881.20 | 1897.20 | 1926.86 | 1922.67 | negative |
| Base model + IBW + ALT | ALT | V | PROP | 1986.88 | 2002.88 | 2032.54 | 2028.35 | negative |
| Base model + IBW + Reflux | Reflux | V | POWER | NA | NA | NA | NA | NA |
| Base model + IBW + Reflux | Reflux | V | EXP | 1877.90 | 1893.90 | 1923.56 | 1919.36 | negative |
| Base model + IBW + Reflux | Reflux | V | PROP | 2020.25 | 2036.25 | 2065.90 | 2061.71 | negative |
| Base model + IBW + MDZ | MDZ | V | ADD | 1914.89 | 1930.89 | 1960.55 | 1956.36 | negative |
| Base model + IBW + MDZ | MDZ | V | PROP | 1913.01 | 1929.01 | 1958.66 | 1954.47 | negative |
| Base model + IBW + MDZ | MDZ | CL | ADD | 1989.15 | 2005.15 | 2034.81 | 2030.62 | negative |
| Base model + IBW + MDZ | MDZ | CL | PROP | 1983.79 | 1999.79 | 2029.45 | 2025.25 | negative |
| Base model + IBW + RRT | RRT | CL | ADD | 2068.42 | 2084.42 | 2114.07 | 2109.88 | negative |
| Base model + IBW + RRT | RRT | CL | PROP | 2021.58 | 2037.58 | 2067.24 | 2063.05 | negative |
| Base model + IBW + RRT | RRT | V | ADD | 1944.16 | 1960.16 | 1989.82 | 1985.63 | negative |
| Base model + IBW + RRT | RRT | V | PROP | 2021.87 | 2037.87 | 2067.53 | 2063.34 | negative |
| Base model + IBW + VPA | VPA | V | ADD | 1986.78 | 2002.78 | 2032.44 | 2028.24 | negative |
| Base model + IBW + VPA | VPA | V | PROP | 2022.96 | 2038.96 | 2068.62 | 2064.43 | negative |
| Base model + IBW + VPA | VPA | CL | ADD | 1986.02 | 2002.02 | 2031.67 | 2027.48 | negative |
| Base model + IBW + VPA | VPA | CL | PROP | 1947.01 | 1963.01 | 1992.67 | 1988.47 | negative |
| **Final model: Base model + IBW (allometric scaling on V and CL)** | | |  | **1813.02** | **1827.02** | **1852.97** | **1848.78** |  |
| COV: Covariate; OFV: Objective function value; AIC: Akaike information criteria; BIC: Bayesian information criteria; BICc: Corrected Bayesian information criteria; V: Volume of distribution; CL: Total body clearance; ADD: Additive function; PROP: Proportional function; POWER: Power function; EXP: Exponential function; ALLO: Allometric scaling; RRT: Renal replacement therapy; TBW: Total body weight; IBW: Ideal body weight; HT: Height; CREA: Serum creatinine; BILI: Bilirubin; AGE: Age; BMI: Body mass index; ALB: Albumin; MDZ: Midazolam; AST: Aspartate aminotransferase; ALT: Alanine aminotransferase; VPA: Valproate. | | | | | | | | |

## Probability of target attainment analysis: C++ model script for “mrgsolve”

$PROB

Model of Stoschus et al. 2025

Description: IV 1 CMT model linear Elimination

Author: Maximilian Stoschus

$CMT @annotated

GUT : Dosing compartment (mg)

CENT: Central PK compartment (mg)

$MAIN

double ka = TVKA;

double F = TVF;

double CL = TVCL * exp(ETACL + IOV) * pow((IBW/68.75), 0.75);

double V1 = TVV * exp(ETA(2)) * (IBW/68.75);

double ke = CL/V1;

double dose = self.amt;

// double C1 = CENT/V1;

D_CENT = 0.0833;

// clearance with iiv and iov

double IOV = ETA_occ1;

if(occ ==2) IOV = ETA_occ2;

if(occ ==3) IOV = ETA_occ3;

if(occ ==4) IOV = ETA_occ4;

if(occ ==5) IOV = ETA_occ5;

if(occ ==6) IOV = ETA_occ6;

if(occ ==7) IOV = ETA_occ7;

if(occ ==8) IOV = ETA_occ8;

if(occ ==9) IOV = ETA_occ9;

if(occ ==10) IOV = ETA_occ10;

if(occ ==11) IOV = ETA_occ11;

if(occ ==12) IOV = ETA_occ12;

if(occ ==13) IOV = ETA_occ13;

if(occ ==14) IOV = ETA_occ14;

$ODE

double C1 = CENT/V1;

dxdt_GUT = -ka * F * GUT;

dxdt_CENT= ka * F * GUT - ke * CENT;

$PARAM @annotated

TVKA: 1.9 : Ka

TVF : 0.96 : Bioavailability

TVCL: 0.38 : Clearance

TVV: 34.3 : Volume of distribution

occ : 1 : default_value_occ

IBW : 70 : Ideal body weight (kg) // Now IBW is adjustable

$OMEGA @annotated

ETACL: 0.4 : ETA on clearance

ETAV: 0.71 : ETA on V1

IOV defined as omega

ETA_occ1: 0.36 : KAPPA on clearance

ETA_occ2: 0.36 : KAPPA on clearance

ETA_occ3: 0.36 : KAPPA on clearance

ETA_occ4: 0.36 : KAPPA on clearance

ETA_occ5: 0.36 : KAPPA on clearance

ETA_occ6: 0.36 : KAPPA on clearance

ETA_occ7: 0.36 : KAPPA on clearance

ETA_occ8: 0.36 : KAPPA on clearance

ETA_occ9: 0.36 : KAPPA on clearance

ETA_occ10: 0.36 : KAPPA on clearance

ETA_occ11: 0.36 : KAPPA on clearance

ETA_occ12: 0.36 : KAPPA on clearance

ETA_occ13: 0.36 : KAPPA on clearance

ETA_occ14: 0.36 : KAPPA on clearance

$SIGMA @annotated

PROP: 0.082 : Proportional error

$TABLE

double CP = C1 + C1 * PROP;

$CAPTURE C1 CP TVF TVKA TVV TVCL ETAV ETACL IOV PROP V1 CL occ dose CMT IBW

# Supplementary figures

## Prediction-corrected visual predictive checks (pcVPC)


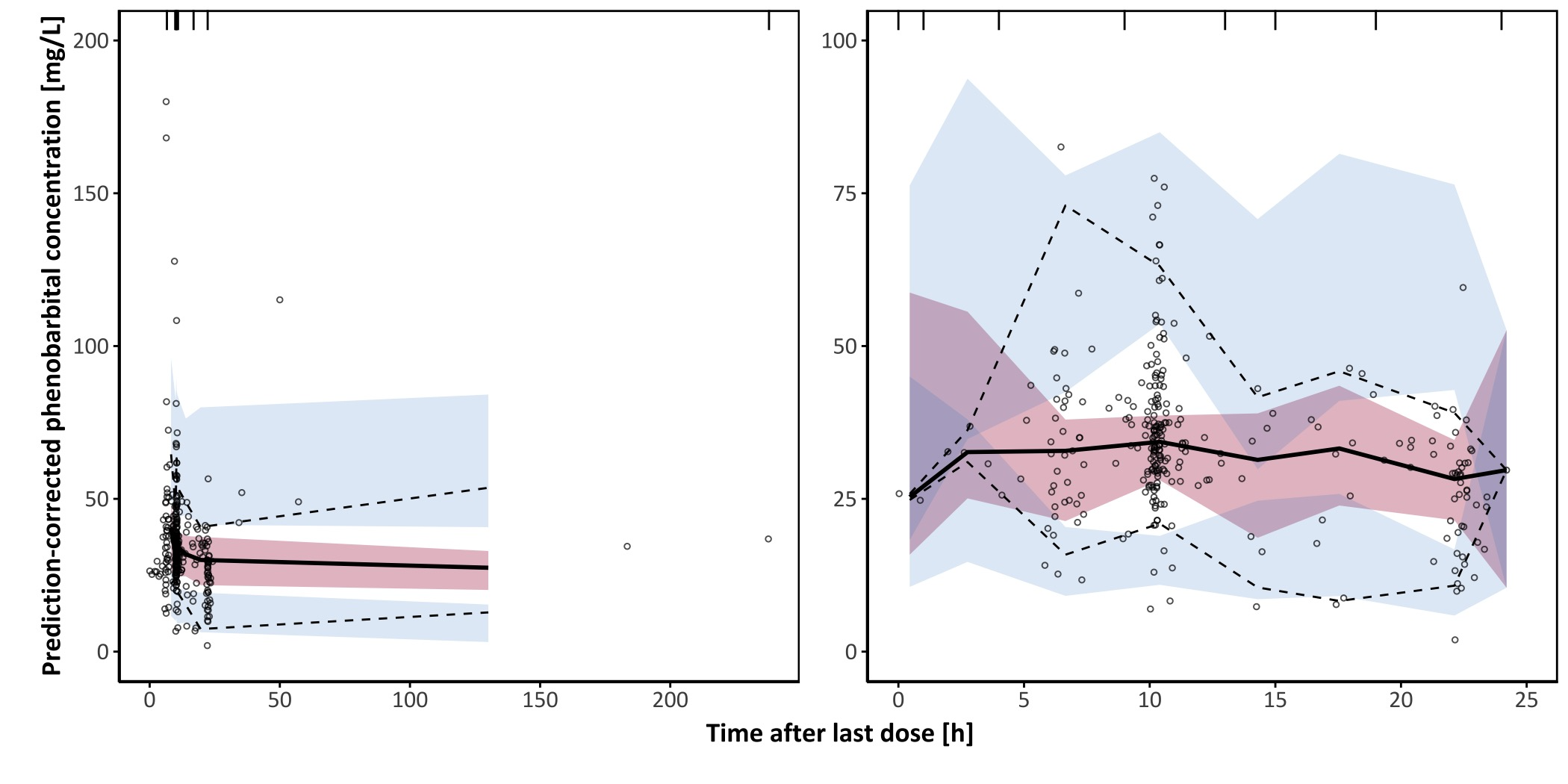


Figure 1: Prediction-corrected visual predictive checks are shown. Solid line represents median of observations and dashed lines represent 5th and 95th percentile. Rings represent observations. 90 percent prediction intervals are shown as the red shaded area for median of observations and blue shaded areas for 5th and 95th percentile of observations. The left graph shows the complete time and concentration range for observations and predictions. The right graph focuses on a smaller time and concentration range where most observations occur.

## Individual concentration-time profiles


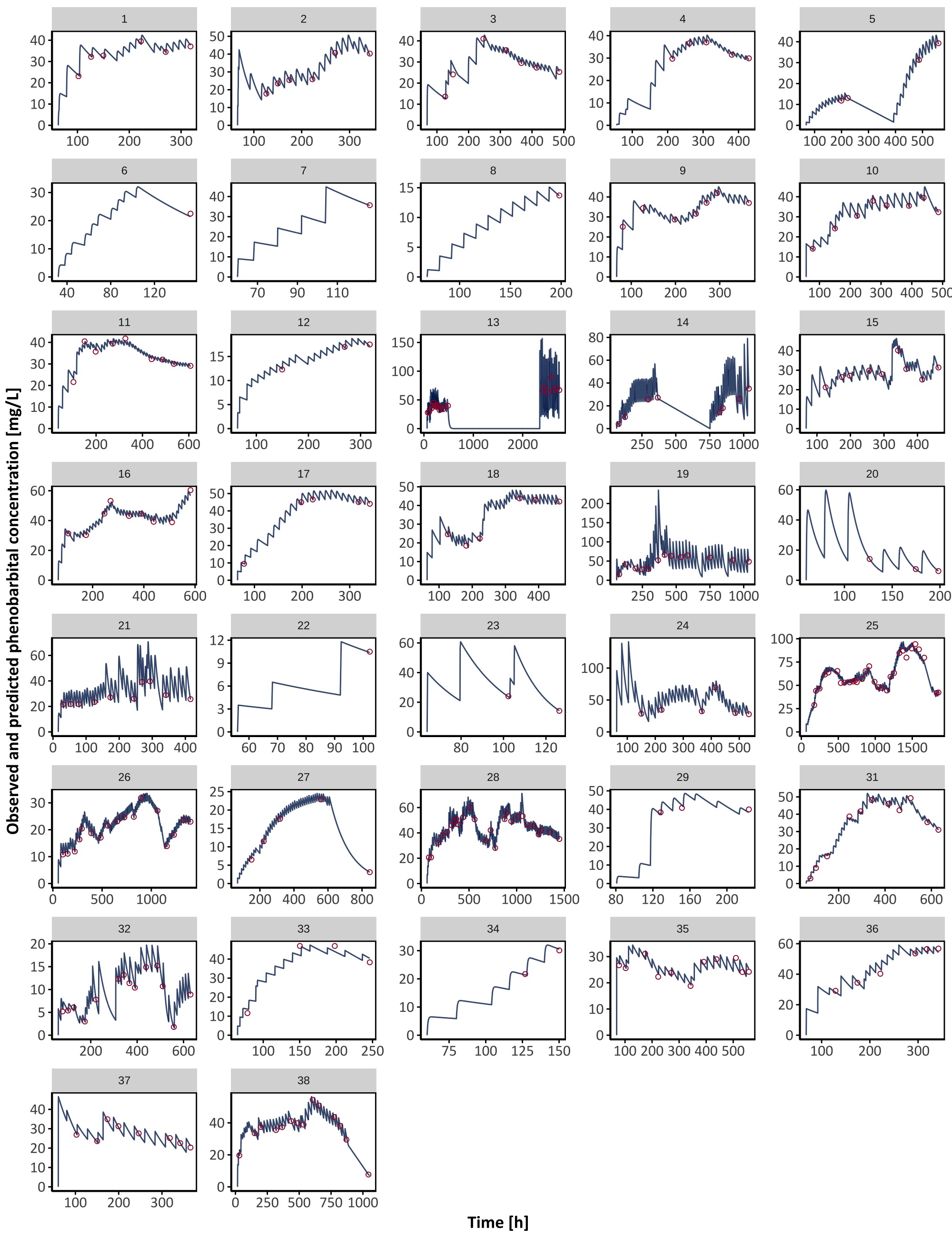


Figure 2: Concentration-time profiles of individual predictions and individual observations for each patient. Lines represent predictions of the final model. Red rings represent observations.

## Concentration-time profiles for simulated 12-hour and 24-hour schedules


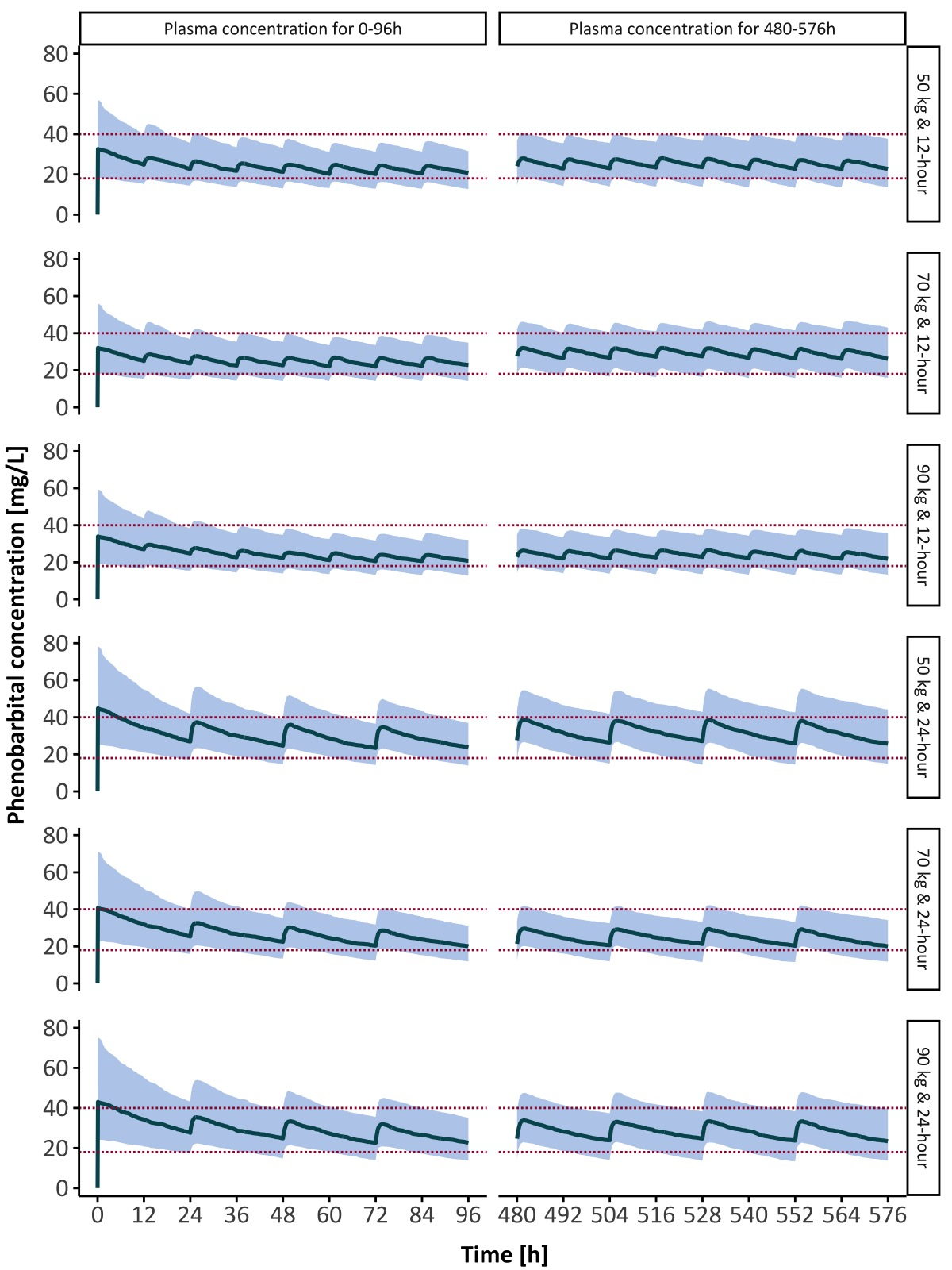


Figure 3: Concentration-time profiles for a 12-hour and 24-hour dosing schedule across different IBWs (50, 70 and 90 kg), simulated for 1000 patients. In this example, loading doses were given intravenously and maintenance doses were given orally. The solid lines represent the median, the blue shaded areas show the 50% prediction interval, and the dotted red lines indicate the target range (18-40 mg/L).
